# Subsequent Waves of Convergent Evolution in SARS-CoV-2 Genes and Proteins

**DOI:** 10.3390/vaccines12080887

**Published:** 2024-08-05

**Authors:** Daniele Focosi, Pietro Giorgio Spezia, Fabrizio Maggi

**Affiliations:** 1 North-Western Tuscany Blood Bank, Pisa University Hospital, 56124 Pisa, Italy; daniele.focosi@gmail.com; 2 Laboratory of Virology and Laboratory of Biosecurity, National Institute of Infectious Diseases Lazzaro Spallanzani—IRCCS, 00149 Rome, Italy; fabrizio.maggi@inmi.it

**Keywords:** COVID-19, SARS-CoV-2, evolution, Spike

## Abstract

Beginning in 2022, following widespread infection and vaccination among the global population, the SARS-CoV-2 virus mainly evolved to evade immunity derived from vaccines and past infections. This review covers the convergent evolution of structural, nonstructural, and accessory proteins in SARS-CoV-2, with a specific look at common mutations found in long-lasting infections that hint at the virus potentially reverting to an enteric sarbecovirus type.

## 1. Introduction

As we pass the four-year mark of the pandemic, our understanding of SARS-CoV-2 continues to evolve, unveiling novel aspects such as the emergence of viral microRNAs [1], their potential for human genome integration [2], their capabilities for compartmentalization [3,4], and their persistence in even immunocompetent individuals [5,6,7,8] that may lead to prolonged symptoms [9,10]. The virus has proven to be panzootic amongst placental mammals [11], with numerous instances of reverse zoonosis already recorded [12,13,14], and with certain variants continuing to persist despite nearing extinction in humans [15,16]. This extensive cross-species transmission fosters substantial chances for divergent evolution. The mutation rate of SARS-CoV-2, currently estimated at 28.4 substitutions annually (https://nextstrain.org/ncov/gisaid/global/all-time?l=clock&m=div, accessed on 1 August 2024), is around ten times faster than that of other long-established human RNA viruses, including Measles, Mumps, Syncytial Respiratory Virus, Dengue virus, Zika virus, West Nile virus, Enterovirus D68, or influenza A(H3N2) virus.

From December 2021 to August 2024, over 4000 sublineages have been designated by the Phylogenetic Assignment of Named Global Outbreak Lineages (PANGOLIN) working group [17], all classified within the World Health Organization’s designation of the Omicron variant as a variant of concern (VOC) [18]. Worldwide studies have demonstrated an increase in serological divergence [19], leading to enhanced immune evasion [20] and, consequently, a gradual rise in the virus’s reproductive number [21]. Notably, the year 2023 marked the first dominance of a recombinant SARS-CoV-2 lineage (designated as XBB, with the XBB.* family consisting of 1000 members), alongside the emergence of various second-generation recombinants [22,23].

Convergent evolution in proteomics is the process where unrelated members of a “variant soup” develop similar amino acid changes due to independent genetic mutations. With the focus on anti-Spike antibodies as a marker for immunity post-infection or vaccination and the Spike protein being the primary antigen in approved vaccines, most convergent evolution research centers on this protein. However, increased viral spread in regions using whole-virus-inactivated vaccines suggests that immune responses to other viral proteins are also influencing SARS-CoV-2’s evolution. While the convergence during the pandemic’s first two years (2020–2021) has been thoroughly reviewed [24,25,26], this review will concentrate on the key structural characteristics influencing the evolution of SARS-CoV-2’s structural, nonstructural, and accessory proteins.

## 2. Convergent Evolution in SARS-CoV-2 Structural Proteins (SP)

The structural proteins (SP) of SARS-CoV-2 include Spike (S), Nucleocapsid (N), Membrane (M), and Envelope (E). Each one will be examined individually in the following sections:

### 2.1. Convergence in the Spike Protein

The Spike protein is composed of 1273 amino acids. Its S1 segment contains amino acids 14 to 685. The N-terminal domain (NTD) stretches from amino acid 14 to 306, while the receptor-binding domain (RBD) is comprised of amino acids 319 to 541. Within this domain, the angiotensin-converting enzyme 2 (ACE2) receptor-binding motif (RBM) is defined by amino acids 437 to 508. The whole Spike protein structure includes one phosphorylation site at T791 and five disulfide bonds: C15-C136 within the NTD and C480-C488, C336-C361, C391-C525, plus C379-C432 within the RBD. For efficient entry into the cytoplasm and subsequent replication, the Spike protein must be cleaved twice. The first cleavage occurs near amino acid 685, the S1/S2 boundary, by furin in the Golgi apparatus [27], and a second cleavage happens near amino acid 816 (S2′ site) [28]. This cleavage is carried out either by TMPRSS2—predominantly found in the lower respiratory tract, which induces cell membrane fusion—or by cathepsins in endosomes. The initially lower incidence of pneumonia in BA.1 cases [29] was thought to be due to a reduced ability to utilize the transmembrane protease serine 2 (TMPRSS2) pathway of cell entry [30,31,32], leading to a greater reliance on the cathepsin pathway [33]. However, this turned out to be a laboratory artifact caused by the overexpression of TMPRSS2 and ACE2 in cell lines, which misrepresented the true dynamics of infection [34,35,36,37,38]. Subsequent studies clarified that TMPRSS2-mediated cleavage of ACE2 significantly boosts replication of both SARS-CoV-1 [39,40] and SARS-CoV-2 [41]. ACE2 primarily functions as an enhancer for the B0AT1 (SLC6A19) or SIT1 (SLC6A20) amino acid transporters across various tissues, including the intestine, cardiomyocytes, and renal proximal tubule epithelial cells, where it also obscures the TMPRSS2 cleavage site [42]. Consequently, these cell types, when derived from corresponding tissues, exhibit lower competence for SARS-CoV-2 infection.

While other human respiratory viruses such as COV-229E [43] or influenza A evolve in clear, ladder-like patterns, or in the case of COV-OC43 [43] or influenza B in two ladder-like trees, SARS-CoV-2 has exhibited a more complex phylodynamic structure [43]. Unlike its predecessors, where each new variant usually arises from the lineage of the previous successful one, SARS-CoV-2’s subsequent variants have not followed this trend, complicating the selection process for vaccines. Additionally, the convergence in the Spike protein merits closer examination due to its potential for significant insights. Foster et al. have recently demonstrated that convergent evolution, as seen with mutations like A67V or H655Y, can also occur in vitro after 33–100 serial passages through four VOCs and three variants under investigation (VUI) in Vero E6 cells, even without the presence of an immune response from a multicellular organism [44].

### 2.2. Convergence in the RBD

**Figure 1:**
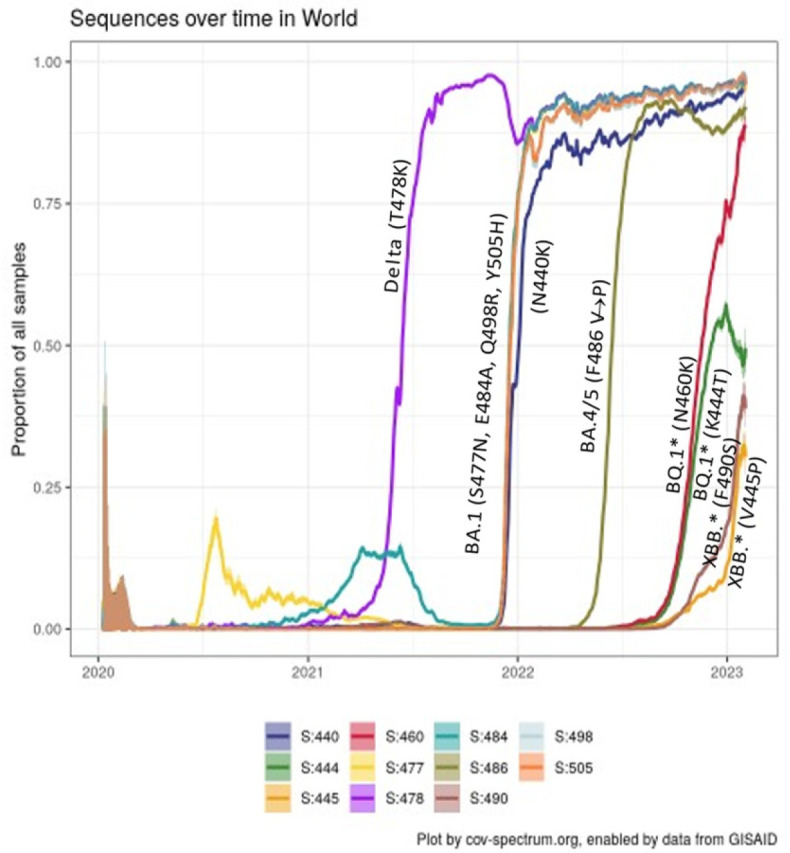
Timeline of RBD Spike protein mutation fixation across SARS-CoV-2 VOCs.

The receptor-binding domain (RBD) of the Spike protein is made up of amino acids that range from 319 to 541, with the RBM spanning amino acids 437 to 508. When observing individual sublineages, there are patterns akin to waves where each new VOC completely overtakes its predecessor (as shown in Figure 1).

However, examining specific mutations reveals that some have become permanently established, irrespective of changing sublineages. Notably, within the RBM, 11 residues (440, 444, 445, 460, 477, 478, 484, 486, 490, 498, and 505), along with an additional 9 within the RBD (339, 368, 371, 373, 375, 376, 405, 408, and 417), have persistently mutated when compared to the original wild-type strain.

**Figure 2:**
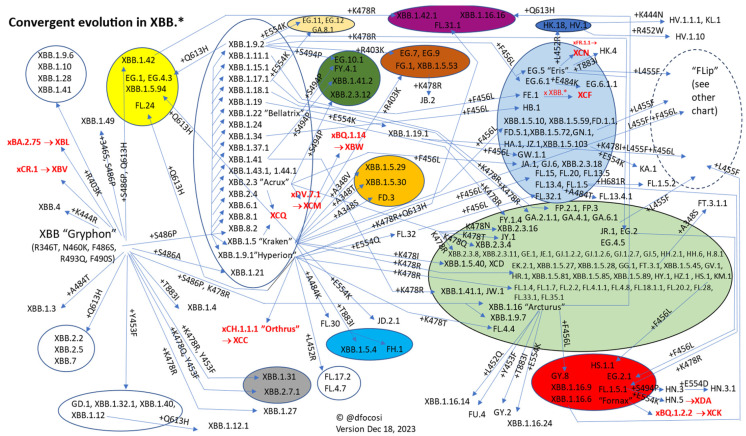
Summary of convergent evolution of Spike protein in XBB.* sublineages.

In 2021, evolutionary trends largely centered on the E484 residue, but by late 2022, convergence mainly involved residues 346, 444, and 486 [20,45]. Early into 2023, it shifted to primarily affect positions 356 (Figure S1), 403 (Figure S2), 445 (Figure S3), 450 (Figure S4), 456 (Figure S5), 478 (Figure S6), 554 (Figure S7), 613 (Figure S8), and 883 (Figure S9). Since spring 2023, the XBB.* variant has prevailed, with its Spike protein evolution depicted in Figure 2.

The mutations 456L and 478R are both recurring and cooperative, enhancing their growth potential together. Remarkably, other residues like K304, which had been constant for three years, started changing within the context of XBB.* + F486P.

**Figure 3:**
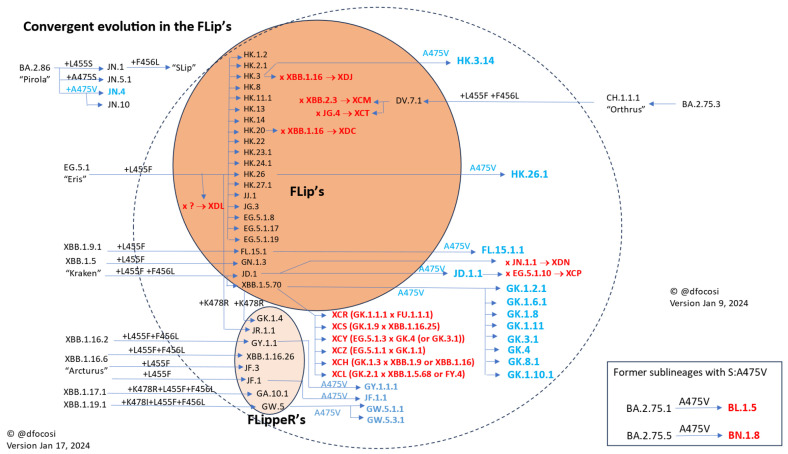
Convergent evolution in FLip’s mutations, adapted from ref. [46].

Serial convergence to L455F first and F456L/V later (a combination nicknamed “FLip” or “SVip”) was among the main phenomena of 2023 [46] (Figure 3). The FLip mutations introduced two additional hydrogen bonds, H34-Q493 (3.57 A) and H34-S494 (2.57 A), which enhanced the affinity of FLip’s RBD to ACE2 while blocking class 1 antibody recognition [47]. FLips has faced notable competition from the Pirola clan since the autumn of 2023 [48]. This is evidenced by JN.1 carrying the related mutation S:L455S, demonstrating the lineage-independent aspect of convergent evolution occurring simultaneously.

**Figure 4:**
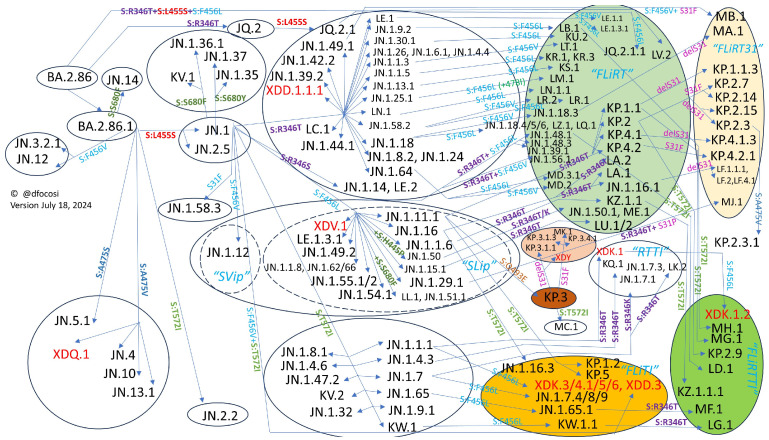
Summary of convergent evolution of Spike protein in JN.1* sublineages.

The pattern of convergent evolution in JN.1*, primarily observed with mutations F456L, A475V, and T572I, is illustrated in Figure 4. In the second four-month period of 2024, the emergence of R346T alongside F456L gave rise to the FLiRT variants (such as KP.2), which quickly became predominant [49]. As of this writing, the FLiRT31 variants (sublineages of FLiRTs that include S:S31del or S:S31F deletions) and the KP.3 lineage with S:Q493E are superseding FLiRTs [50]. The mutation S:Q493E represents one of the most infrequent nucleotide changes (C to G): Q493K is prevalent among cryptic variants and was selected for in murine models [51,52], while Q493A and Q493V have been identified in several cases of chronic infection.

**Figure 5:**
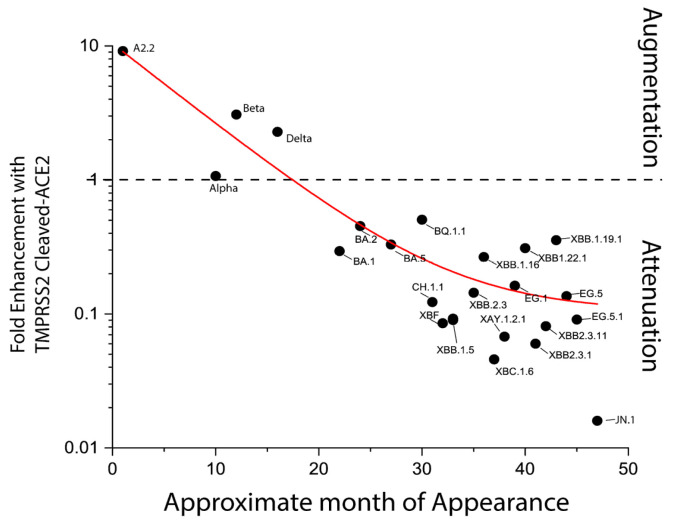
The trends towards declining fold-enhancement in infectivity with TMPRSS2-cleaved ACE2 in time. Sourced from ref. [41].

The decrease in ACE2 affinity caused by Q493E can be reversed in the presence of L455S + F456L, as observed in BA.2.86 and in XBB.15 [53]. These compensatory mutations significantly increase ACE2 affinity and re-establish acidity in the unusually basic RBM, which includes additional mutations like V445H, N481K, and A484K in BA.2.86. Figure 5 delineates how alterations in the Spike protein incrementally dampened the efficacy of TMPRSS2-mediated cleavage of ACE2 over time.

### 2.3. Epistatic Interactions among Spike Mutations

Epistatic interactions may either enhance ACE2 binding (permitting additional RBD mutations such as Q498R-N501Y in Omicron [54,55]) or reduce it (hindering further RBD changes like L452R-T478K). Co-occurring pairs like R408S-D405N are also observed, notably in BA.2/4/5* variants.

A fascinating aspect of SARS-CoV-2’s evolution involves exclusive occurrences among the top three ACE2 affinity-enhancing mutations (R403K, Y453F, and S486P) and the top three disrupting mutations (Q498H and N501Y), with rarities like FG.1 and XBB.1.41 exhibiting both R403K and S486P, compensated by the ACE2-affinity-reducing mutation L513F. Additionally, a unique mutual exclusion exists between R346X and N450D; interestingly, this combination materialized only in FLiRT’s. Notably, when LB.1.2.1 was recombined with KP.3.2 FLiRT to create XDY, R346T disappeared. However, it is important to recognize that specific mutations’ effects are often epistatic, meaning they become apparent only within certain sublineages—for instance, R403K does not increase ACE2 affinity against the B.1* lineage background [56].

### 2.4. “Yo-Yo” and Fixated Mutations

It has become apparent that several residues of the Spike protein are subject to cyclical mutation and reversion. As of December 2023, unmutated residues account for the majority of the RBD (196/223, 86%) [57]. RBD mutations were fixated at 20 amino acid positions (11 within and 9 outside the RBM): residue 478 mutated before Omicron, while 13 mutated residues were fixated since BA.1 (339, 371, 373, 375, 376, 405, 408, 417, 440, 477, 484, 498, and 505), and the other 6 residues mutated with ongoing Omicron evolution (368, 445, 460, 486, 490, and recently 456). “Yo-yo” residues (i.e., those mutating and reverting) were only 7 (444, 446, 452, 493, 496, and 501, plus 346 within the RBM) (Figure 1). Namely, positions 444, 492, and 493 had 2 sequential changes, 346, 446, and 501 had 3 changes, and 452 had 4 changes. After December 2023, the pendulum has already switched somewhat with JN.1* (e.g., L452W and Q493E in KP.3). It should be noted that 2-nucleotide changes are generally a hallmark of immune pressures, but only a few of them occurred during Omicron evolution, such as V445H and L452W (recently popped out in BA.2.86) or, much more diffusely, A484K and F486P.

### 2.5. Disappearance of the Furin-Cleavage Site (FCS)

The Furin Cleavage Site (FCS) at amino acids 681–685 activates the Spike protein for cell entry by enabling furin-mediated cleavage and facilitating membrane fusion, which requires an additional cut by TMPRSS2. This step is crucial for the virus to infect pneumocytes and spread among ferrets [58]. Mutations like P681H/R and S:N679K have increased its effectiveness, especially in Omicron. Significantly, mutations that disrupt the FCS, such as S:R683W/Q, S:K679M, S:S680F, or S:R681P, have emerged frequently in the JN.1 variant in the absence of compensatory mutations without significantly disrupting viral fitness.

### 2.6. Saltation Variants

Many Omicron saltation variants have been detected in 2023, e.g., BA.2.83, DD.1, BP.1, BA.2.10.4, BA.2.87, and many more undesignated linages (e.g., PANGOLIN issues#1053), among which only BA.2.75 and BA.2.86 have been fit enough to cause a wave through their descendants. The main requisite for success seems to be the initial ACE2 affinity, with incredibly high affinities leaving room for further immune escape (but ACE2 disrupting) mutations (e.g., L455S). High initial affinity is mandatory but can be vinified by other mutations (e.g., intact ORF8 reduces surface Spike expression [59] in XBB.2.3). Saltations are more likely to emerge after persistent infections in immunocompromised hosts. e.g., BA.1, BA.2, BA.4, BA.5, and BA.2.86 (plus an undesignated BA.2.15 derivative) have all likely emerged in the Limpopo and Gauteng provinces of South Africa, known for the highest HIV prevalence in the world (10–25% [60]), with BA.2.87 emerging > 1.5 years after BA.2.

### 2.7. Alterations at N-Glycosylation Sites

N-glycosylation sites consist of N-X-[S/T] motifs (where X is any amino acid other than P). Several mutations create new N-glycosylation sites, which are important to hide viral epitopes from neutralizing antibodies, e.g.,

• S:F32S (convergent in chronics) leads to a N-glycosylation site at N30
• S:R190T (found in Gamma and KP.3.1.1) leads to a N-glycosylation site at N188
• S:H245N (found in BA.2.86) leads to a N-glycosylation site at N245
• S:Y248N (found in BA.2.76 and BA.2.86) leads to a N-glycosylation site at 250
• S:K356T (e.g., in BA.2.86*) leads to a N-glycosylation site at N354. Glycosylation at N354 is a regulatory element for the RBD conformation that lessens viral infectivity.

The decrease in infectivity can be reversed with heparin sulfate, which acts on the “N354 pocket” to facilitate the conformational shift to a “RBD-up” state, thus offering modifiable infectivity. Additionally, N354 glycosylation enhances Spike protein cleavage and cell-to-cell fusion and notably evades a specific group of antibodies associated with antibody-dependent cellular cytotoxicity (ADCC). Coupled with diminished immunogenicity against a backdrop of hybrid immunity, this suggests that glycosylation of a single spike protein amino acid offers a competitive advantage for the virus in human hosts by employing various strategies [61].

On the contrary, other mutations (e.g., common in KP.3) disrupt N-glycosylation sites, e.g., T22N, T29N, or DS31/S31F.

### 2.8. NTD Deletions and Their Functional Consequences

The N-terminal domain (NTD) is perhaps the most mutable region of the SARS-CoV-2 genome. This domain includes five loops designated as N1 (amino acids 14–26), N2 (amino acids 67–81), N3 (amino acids 140–158), N4 (amino acids 174–188), and N5 (amino acids 241–263) [62], with the combination of N1, N3, and N5 creating the noted NTD antigenic supersite [63]. Compared to SARS-CoV-1 and the coronavirus found in Guangdong pangolins, SARS-CoV-2 has significantly longer N2, N3, and N5 loops, which have been hotspots for deletions thus far—for instance, the Δ69-70 deletion in Alpha, BA.1, and BA.4/5 variants’ N2 loops [62]. Deletions and insertions that disrupt disulfide bonds within the NTD are frequently observed in prolonged infections [64], assisting in antibody evasion [65], as seen in other sarbecoviruses. These modifications can also augment the fusion capabilities of SARS-CoV-2 with cell membranes, enhance cellular infection rates, and increase syncytia formation, as was demonstrated when SARS-CoV-2′s NTD was substituted by those from SARS-CoV-1 or the Guangdong pangolin coronavirus [66,67]. However, these alterations come with a cost: Spike protein instability leads to the loss of the S1 subunit and permanent inactivation [67], which only became feasible after the D614G mutation fortified the overall Spike protein structure. Regardless, the fusogenic potential is countered by the S2 portion, notably the N969K mutation [35], which—due to its retention in the Omicron variant—has thus far avoided triggering a Delta variant-like surge.

Several designated sublineages have exploited different strategies to disrupt the disulfide bond between residues C15 and C136:

• Alpha and BA.1.1;
• B.1.640* from France and North Africa had both S:P9L (which, similarly to S12P and S13I, causes cleavage at C15 instead of Q14 within the endoplasmic reticulum [68]) and Δ136-144 [69];
• C.1.2 from Africa similarly had both P9L and C136F;
• B.1.427 (VOI Epsilon) from US West had S13I, but a new disulfide bond with C136 was introduced by W152C, causing total rearrangement and immune evasion [70];
• BA.2.87 had Δ14-22 and Δ135-146.

Replacement of the C15–C136 bond with C15–C258 C248–C258 has been observed in several Alpha sequences in deer and in a single BA.2.86 sequence with G447C + G496C putatively created a fifth disulfide bond within the RBD (the standard four being C480–C488, C336–C361, C391–C525 and C379–C432).

### 2.9. Reversion to SARS-CoV-1 and/or bat-CoV Residues

SARS-CoV-2 is thought to have arisen from a yet-undefined enteric bat sarbecovirus. JN.1* harbors mutations at S:V127, S:F157, S:A570, and S:T572 (as for bat-CoV and SARS-CoV-1): furthermore JN.1* shares R403K, F456L, ΔVE483-484, T478K, P621S, and D796Y with SARS-CoV-1, and K356T, N440K, and L455S with bat coronavirus. BA.2.87 similarly has D215G and N460K.

Notably, JN.1 also has S:S50L, a formerly extremely rare mutation whose reversion reduced infectivity in CaLu-3 cells [71]. S:S50L was more prevalent in GI tract swabs than in NPS in China and Hong Kong in Spring 2020 (13% vs. 1.5%) and led to the flexibility of the S1 subunit [72]. Notably, gut-tropic bat sarbecoviruses have both S:H49Y and S:S50L, and this could account for the increased fecal shedding seen with JN.1 [72].

### 2.10. Truncation and Mutations of Nucleocapsid

The coronavirus nucleocapsid (N) protein dimer is by far the most abundant protein produced by SARS-CoV-2, and all VOCs have further increased N protein expression levels [73,74]. N consists of 5 domains, 3 IDRs (N/IDR, LKR, and C/IDR), and 2 structural domains (NTD and CTD). For virtually all (+)-sense RNA viruses, actual virions are not necessary to establish infection because genomic RNA (gRNA) is fully infectious, but instead, coronaviruses also need N [75,76]. The N protein plays several roles, such as encapsulating the viral genome [77], working as a viral chaperone by engaging with Nsp3 to aid in viral RNA transcription/synthesis and its stabilization [78,79,80], evading immune responses by associating with viral RNA [81] and disrupting stress granules [82,83], managing gRNA and sgRNA production [84], and virion assembly through phosphorylated residues [85], as well as interactions with M. While NTD, LKR, and CTD can all bind viral RNA, only the CTD is essential for double-stranded RNA binding. Both LKR and CTD are key in impeding SG formation and preventing PKR activation by poly(I·C) [86]. Additionally, the SR-rich domain of N (176–206) binds NSP3’s first Ubl1 domain [87,88].

Phosphorylation of N:S206 by cellular SPRK1/2 prompts a 6-aminoacid phosphorylation chain reaction by GSK3 (S186, S190, S194, and more) [89]. This reduces N binding to RNA and, therefore, stifles encapsidation and enhances NSP3-N interaction [78,87]. Several SARS-CoV-2 variants of concern carry N protein mutations (e.g., N:S202 or N:S206I) that reduce phosphorylation and enhance the efficiency of viral packaging. By keeping RNA in a loose, accessible conformation, as opposed to the knotty, compressed vRNP-packaging form, pN facilitates viral RNA transcription. N also unwinds TRS RNA, enhancing the template switching required for sgRNA transcription [90]. It leads to the recruitment of cellular helicase DDX1, which assists in the production of gRNA and long sgRNAs (Spike, ORF3a, E, and M) at the expense of short sgRNA (ORF7a, ORF8, N, and ORF9b) [85]. In several studies, N:S194L was the main mutation associated with more severe clinical courses [90,91].

Variants of the B.1.1 viral lineage also encode a novel subgenomic mRNA (generated by copy-choice recombination), which translates into a truncated N protein, termed N* or Δ(1-209) or N:210-249, that mediates genome packaging despite lacking the N-terminal RNA-binding domain and SR region [92]. A 3-nucleotide mutation in N created a new transcriptional regulatory sequence (TRS) body (R203K/G204R) in B.1.1 [93], Alpha, Gamma, and Omicron, leading to production of N*, which in turn acquires the ability to bound the human PAF complex (PAF1, LEO1, IWS1, WRD61, CDC73, and CTR9) [94] and leads to higher viral loads [95]: this variant is now universal except for in Delta–Omicron recombinants (dubbed “Deltacrons”) like the XBC lineage and increases infectivity, fitness, and virulence [96].

Importantly, deletions and mutations in N can affect the sensitivity of both rapid antigen tests (RATs, generally based on N-antigen) or cause N-gene target failure (NGTF) in multiplex PCR. Mutants in R203K, G204R, A208G, and M234I found in B.1.1.318 led to NGTF in Allplex, Viasure, and GeneFinder PCRs [97]. Later, this occurred with the ERS31-33 deletion (nucleotide 28362-28370 del) in Omicron [98]. NGTF was proven specific for Alpha-positive samples (N:Δ6-10) (8), AY.4 (N:641∆6 mutation resulting in a two-amino acid deletion of G214 and G215) (9), and BA.2.86* (N > Q229K) using the Allplex™ PCR assay.

As for the Spike protein, reversion to N mutations typical of bat coronaviruses (e.g., N:S37P) has been found in chronics (e.g., in a BN.1.3 saltation reported in April 2024).

## 3. Convergent Evolution in Nonstructural Proteins (NSPs)

The 16 NSPs stem from the ORF1ab gene and make up the first 2/3 of the genome. Most of them are primarily involved in viral replication (reviewed in [99,100,101]). XBB.1.9.1 and XBB.1.9.2 share ORF1a:G1819S and ORF1a:T4175I.

NSP3 is a massive protein, easily the largest in SARS-CoV-2, and has 16 domains (macrodomains (Mac)1, Mac2/SUD-N, Mac3/SUD-M, DPUP/SUD-C, PLPro, and Ubl2 at the amino-terminus). PLPro is among the most notable domains. ORF1a:K1795Q (a.k.a. NSP3:K977Q or PLPro K232Q) has been one of the commonest cryptic lineages reported from immunocompromised patients harboring persisting infections (5–10%) and in cryptic lineages from wastewaters (>50%, likely representing the most extreme persisting infections), while rare in circulating sublineages (e.g., in Gamma and BS.1), so it can be used for “carbon dating” the infection. K1795Q falls within the papain-like protease (PLPro) domain of NSP3 and enhances poly-ubiquitin cleavage, enhancing viral immune evasion [102,103]. NSP3, NSP4, and NSP6 cooperate to form double-membrane vesicles (DMV) out of the endoplasmic reticulum that serve to shield dsRNA formed during SARS-CoV-2 transcription/replication from the immune system. The RNA reaches the cytoplasm via pores in the DMV constituted by NSP3 and NSP4 [104,105,106], and it is in the DMV that NSP3 interacts with the SR-IDR [87,107], Ubl1 [108], and likely other domains [109] of N. The NTD of the Mac2 domain binds to the cell translation factor Paip1, enhancing its affinity for PAPB (another translation factor) and resulting in a 3-protein complex that enhances the production of viral but not cellular mRNA [110]. The latter is achieved thanks to NSP1, which degrades host mRNA. Without Mac2-Paip1 interaction, viral replication falls by 65–90%.

## 4. Convergent Evolution in Accessory Proteins (AP)

Accessory proteins (AP, i.e., ORF3a, ORF6, ORF7a, ORF8, ORF9b, and N*) are all encoded by subgenomic mRNAs, are less essential than SPs and NSPs, and are mainly involved in interferon antagonism and immune evasion [111]. Several lines at the end of 2023 had an entire deletion of ORF7a, ORF7b, and ORF8; they were GE.1.2.1, GW.5.1.1, FW.1.1, and JP.1.1.

### 4.1. ORF6 Mutations

ORF6:D61L is a TRS-related 3-nucleotide mutation that almost certainly arose due to a copy-choice recombination event and hobbled ORF6. ORF6:D61L creates extended homology for the ORF7a TRS, which should increase its expression. When ORF6:D61L is present (i.e., in BA.4 and all BA.2-derived lineages, including XBB* and JN.*), there is a strong selection against mutations that knock out ORF7a, suggesting they may have overlapping functions. ORF6:D61L impairs ORF6′s immune evasion function by reducing its ability to block cellular trafficking in and out of the nucleus [112], and this could have contributed to BA.5 outperforming BA.4 despite an identical Spike and time of emergence. ORF6:D61L is instead usually dropped in successful recombinants between BA.2-derived and non-BA.2 lineages (e.g., XAY, XBC, and XBJ), recombination being the most likely way to revert a 3-nucleotide mutation.

### 4.2. ORF7a Loss

As stated above, ORF7a is likely redundant with ORF6, and the proteins tend to be mutually excluded. Alpha first eliminated ORF7 expression, and the elimination of ORF7 (in non-ORF6:D61L lineages) represents a trend in SARS-CoV-2 evolution. C27389T marks the TRS for ORF7a in the exact same way C288889T spoils the ORF8 TRS: both alter the ideal core TRS sequence (AAACGAAC) to a much less optimal one (AAATGAAC). C27389T has tended to grow with all sublineages but not in BA.2-derived lineages (such as XBB and JN.1) because of the occurrence of ORF6:D61L. ORF7a:H47Y in BF.7.14 drove the winter 2022–2023 China wave [113]. The role of that mutation remains uncharacterized, but the close ORF7a:F59 is known to down-regulate HLA class I [114]. The H47Y mutation has the effect of impairing the ability of SARS-CoV-2 ORF7a to oppose the type-I interferon (IFN-I) response and to downregulate Major Histocompatibility Complex-I (MHC-I) cell surface levels. However, it has no effect on its anti-SERINC5 function [115].

### 4.3. ORF7b Stop Codons Create New Proteins

ORF7b is the smallest protein in SARS-CoV-2. In May 2023, it was noted that two non-XBB but highly fit competitors, namely XBC.1 and FR.1, had relatively similar stop codons. In XBC.1.*, the 2-nucleotide deletion in codon 13 causes a stop at codon 32-33. In FR.1, similarly, there is a 1-nucleotide insertion at codon 13 that causes an identical stop codon. The resulting protein lacks the final 11 polar amino acid residues, but the frameshift has caused 7 out of the first 14 residues to turn polar.

### 4.4. ORF8 Deletion

The proposed functions of ORF8 include acting as a virokine, which mimics a viral cytokine [116], suppressing the expression of host MHC [117,118], reducing Spike protein expression on the cell surface [59], possessing histone-like activity [119], regulating interferon [120], initiating endoplasmic reticulum stress responses [121], and triggering inflammation [122]. Nevertheless, at least some of these activities persist in strains lacking ORF8 expression, suggesting a high level of redundancy between SARS-CoV-2 proteins [118]. It was initially observed that, beginning with the VOC Alpha (ORF8:Q27*STOP), SARS-CoV-2 variants have developed mutations leading to decreased similarity in the ORF8 transcription regulatory sequence. The BA.1.1 lineage completely ceased ORF8 expression. C27889T is found in nearly all BA.5.* sublineage sequences, while G27890T is found in XBC.* sublineages. Both C27889T and G27890T downregulate or cancel ORF8 subgenomic mRNA expression. The most abundant examples of ORF8 silencing are XBB.1* sublineages with ORF8:G8*STOP (ORF8 truncated before the end of the signal peptide is nonfunctional). By June 2023, fewer than 10% of the lineages in circulation are expected to possess a complete ORF8 [123]. Research by Tamura and colleagues demonstrated that the XBB.1.5 lineage’s ORF8 nonsense mutation impairs MHC expression [124]. Notably, the XBB.2.3 lineage still retains an intact ORF8.

### 4.5. ORF9b Increased Translation

ORF9b originates from the same genetic sequence as the N protein but utilizes a different reading frame, causing ribosomes to translate ORF9b infrequently during “leaky scanning”. It was observed that ORF9b/N acquired a second translational regulatory signal, improved by specific mutations like C28311T and C23312T, particularly in the Gamma variant with an extra-overlapping TRS induced by a four-nucleotide insertion. The Alpha variant’s translation of ORF9b was further enhanced by the mutation GAT28280CTA (N:D3L) [125,126]. Conversely, alterations such as the A28271 deletion in Delta and Alpha—or the A28271 mutation in Omicron—deteriorated the Kozak sequence for N. XBB.1.9 variants, outpacing XBB.1.5 due to the ORF9b:I5T mutation (T28297C), have shown competition despite their effects remaining unclear; this mutation, along with D89E, has also emerged in patients with prolonged infections.

## 5. Shared Mutations in Persistent Infections

Patients with compromised immune systems have lower immune pressures, leading to a silent mutation rate of less than 10%, unlike the higher rates of over 60% observed in bats and white-tailed deer [127] and approximately 25% in the wider human population. There is also evidence of convergent evolution within hosts during persistent infections. Mutations in chronic cases are primarily non-synonymous and often result from multiple simultaneous 2- or 3-nucleotide mutations. In contrast, more than 99.9% of mutations in the broader community are typically single-nucleotide variants. Common mutations found in chronic patients and specific sublineages include L452R/K, N460K, S477N, T478K, N481K, E484A, and T791I, the latter disrupting the sole phosphorylation site on the Spike protein [128]. Less frequently, mutations such as S:F32S, S:D339H, S:L368I, S:R408T, S:K417T (also seen in BA.2.87.1 and Gamma but less common than S:K417N), S:K440R, S:Q493K (contrasted with S:Q493R), S:Q498H and S:Q498Y (often combined with S:N501S/T, but not the highly prevalent S:N501Y), deletions of two amino acids in the region of residues 440–448, deletions of one amino acid between residues 483–484, reversion of V486F, and variations at position 796 and 936 like Y796H and D936H/Y/N/G/E/V/T are observed [129].

There are also uncommon mutations outside of the Spike protein, such as E:T11A, E:T30I [130], M:A104V, M:H125Y [130], N:L13P [83] reversion, and various ORF1a mutations like K1795Q, I3244T, and T4175I, plus mutations in ORF9b and ORF8 [131]. These unique changes act as markers to trace variants originating from prolonged infections in immunocompromised patients—for instance, BA.2.86 with the E483del mutation. Interestingly, some mutations conducive to surviving within a host prove unfavorable for spreading between hosts, as they’re absent from prevalent strains—for example, E:T30I and M:H125Y.

Approximately one-fourth of cryptic genetic lineages exhibit two specific mutations, C25162A and C125163A, within the open reading frame (ORF). Although the first mutation is synonymous and does not alter the protein sequence, the second leads to an amino acid substitution, Q1201K. It is observed that over 60% of lineages that acquire the initial mutation also develop the subsequent one. Together, these modifications create a Transcription Regulatory Sequence (TRS) that results in the formation of a novel subgenomic RNA. Within the Omicron variant, the N:VTQ270-272LIL alterations coincide with changes at ORF1a:4395 (NSP12_3-6), ORF1b:820-824 (NSP12_829-833), and additions like ORF1:N1540K/S, T1542I, and T1543I (NSP3_N722K/S, T724I, and T725I). The final mutation is situated in a domain before Ubl2 and PLpro (DPUP), previously referred to as the SARS-unique domain C (SUD-C). In studies involving K18-ACE2 mice, sequential infections using Beta or Delta variants led to the emergence of the S:S371F and S:1182G mutations. These mutations are associated with interactions with mammal-specific GOLGA7 and ZDHHC5 proteins, which play roles in viral cell entry and antiviral defense mechanisms. Despite a marked increase in disease severity from late-passage viruses, there was a concurrent decrease in Spike protein expression attributable to the C21557T mutation, which disrupts TRS [132].

Monitoring of cryptic lineages is often performed in wastewater, where contamination by small mammals is hard to exclude. When comparing the current consensus SARS-CoV-2 genome to bat sarbecoviruses, it is clear that mutations occurred at the same residues that are found reverted in cryptic lineages. Prominently in ORF1ab (V38A, S376P, V1393M, F1779L, K1795Q, T1882I, A3143V, L3606V, F6710S, F6715L), Spike (F32S, S50L, T76I, R346T, A372T (which restores glycosylation site), Q498H/Y, H519N), ORF3s (I10L), N (S37P), and S2m (T29758G).

## 6. Forecasting SARS-CoV-2 Evolution

The evolution of the SARS-CoV-2 Spike protein has generally balanced maintaining an adequate affinity for ACE2 in the RBD with evading neutralizing antibodies induced by previous infections and vaccinations. Deep mutational scanning has been implemented to experimentally quantify the impact of every single possible amino acid change and single-codon deletion on ACE2-binding affinity. This analysis helps predict how Spike protein evolution is constrained by existing mutations, known as epistasis. Such assessments have been conducted for various lineages, including Wuhan-Hu-1 [133], Alpha [54], Beta [54], Delta [54], Eta [54], BA.1, BA.2, and XBB.1.5 [134], with the findings accessible on interactive platforms (https://jbloomlab.github.io/SARS-CoV-2-RBD_DMS_Omicron/RBD-heatmaps, accessed on 1 August 2024 and https://dms-vep.org/SARS-CoV-2_XBB.1.5_spike_DMS/htmls/summary_overlaid.html, accessed on 1 August 2024). Key epistatic changes primarily stem from the existence (Alpha and Beta) or nonexistence (Delta and Eta) of the S:N501Y mutation [54], influencing several residues critical for structure and function. The combined effect of Q498R + N501Y mutations, initially identified in directed evolution experiments [135] and later observed in BA.1 and BA.2 variants, significantly enhances ACE2 affinity, which is necessary for effective immune escape. Additionally, resources to estimate the effects of single amino acid variations on the fitness of any SARS-CoV-2 protein are made available at https://jbloomlab.github.io/SARS2-mut-fitness, accessed on 1 August 2023 [136].

## 7. Conclusions

Several mechanisms are showing increasing trends, such as creating novel TRS that lead to the emergence of new subgenomic mRNAs. This is usually accomplished by copy-choice recombination protein (e.g., N*) but rarely happens with stepwise mutations (e.g., ORF1b:P215L-R216N [137]).

Copy-choice recombination can also be beneficial by either changing 1 or more amino acids that require > 1 nucleotide mutation (e.g., JN.1.5′s ORF1b:V1271T, Alpha’s N:D3L, or BA.2′s ORF6:D61L).

ACE2 is no longer the lone receptor for SARS-CoV-2: many more contributing factors have been identified, such as TMPRSS2, furin, CD147, C-type lectin receptors (CD169, CD209, CD299), neuropilin-1, ASGR2, and KREMEN1, whose expression has been investigated in different human cell types [138].

SARS-CoV-2 variant analysis is quickly moving from manual seeking to automated pipelines (e.g., AUTOLIN [139] or RIVET for recombinants [140]). Tools have been created to infer their impact on the efficacy of antivirals (especially anti-Spike monoclonal antibodies [141]) and to estimate the growth advantage of emerging variants from genomic surveillance (e.g., CoV-Spectrum collection#42 (https://cov-spectrum.org/collections/42, accessed on 1 August 2024) or NextStrain SARS-CoV-2 Forecasts (https://nextstrain.org/sars-cov-2/forecasts/, accessed on 1 August 2024)). Importantly, no difference in risk of hospitalization has been reported so far for XBB.* [142] or JN.* sublineages.

Keeping high levels of genomic surveillance is fundamental to informing public health choices. e.g., vaccine manufacturers have been recently solicited by the WHO to replace the wild-type SARS-CoV-2 lineage with an XBB.* lineage. With a plethora of highly fit XBB.* sublineages occurring right now, forecasting which one will lead the pack at the time of vaccine deployment proves a difficult exercise. We suggest instead focusing on converging Spike mutations and eventually turning back to traditional vaccine manufacturing platforms. In fact, one of the concerns for the near future is that the forecast of much-reduced vaccine deployment will cause a loss of interest in COVID-19 vaccines from vaccine manufacturers, and the few candidates that will hit the market could have prohibitive prices.

## Supplementary Materials

The following supporting information can be downloaded at: https://www.mdpi.com/article/10.3390/vaccines12080887/s1, Figure S1: Summary of independent S:K356T acquisition events in designated PANGOLIN lineages; Figure S2: Summary of S:R403K acquisition events in designated PANGOLIN lineages; Figure S3: Summary of independent S:V445A acquisition events in designated PANGOLIN lineages; Figure S4: Summary of independent S:N450D acquisition events in designated PANGOLIN lineages; Figure S5: Summary of independent S:F456L acquisition events in designated PANGOLIN lineages; Figure S6: Summary of independent S:K478X acquisition events in designated PANGOLIN lineages; Figure S7: Summary of independent S:E554K acquisition events in designated PANGOLIN lineages; Figure S8: Summary of independent S:Q613H acquisition events in designated PANGOLIN lineages; Figure S9: Summary of independent S:T883I acquisition events in designated PANGOLIN lineages.
