# Supplementary material for: Subsequent Waves of Convergent Evolution in SARS-CoV-2 Genes and Proteins
Source: Vaccines (Basel). 2024 Aug 5;12(8):887. doi: 10.3390/vaccines12080887 (PMC11358953; doi:10.3390/vaccines12080887)
Supplement: Supplementary file 1 [file vaccines-12-00887-s001.zip › vaccines-3141163-supplementary.pdf]

Supplementary Materials for

Subsequent Waves of Convergent Evolution in SARS-CoV-2 Genes and Proteins

Daniele Focosi [1], Pietro Giorgio Spezia [2,*] and Fabrizio Maggi [2]

[1] North-Western Tuscany Blood Bank, Pisa University Hospital, 56124 Pisa, Italy; daniele.focosi@gmail.com
[2] Laboratory of Virology and Laboratory of Biosecurity, National Institute of Infectious Diseases Lazzaro Spallanzani—IRCCS, 00149 Rome, Italy; fabrizio.maggi@inmi.it
* Correspondence: pietro.spezia@inmi.it; Tel.: +39-06-55170654

This file includes:

Figures S1–S9

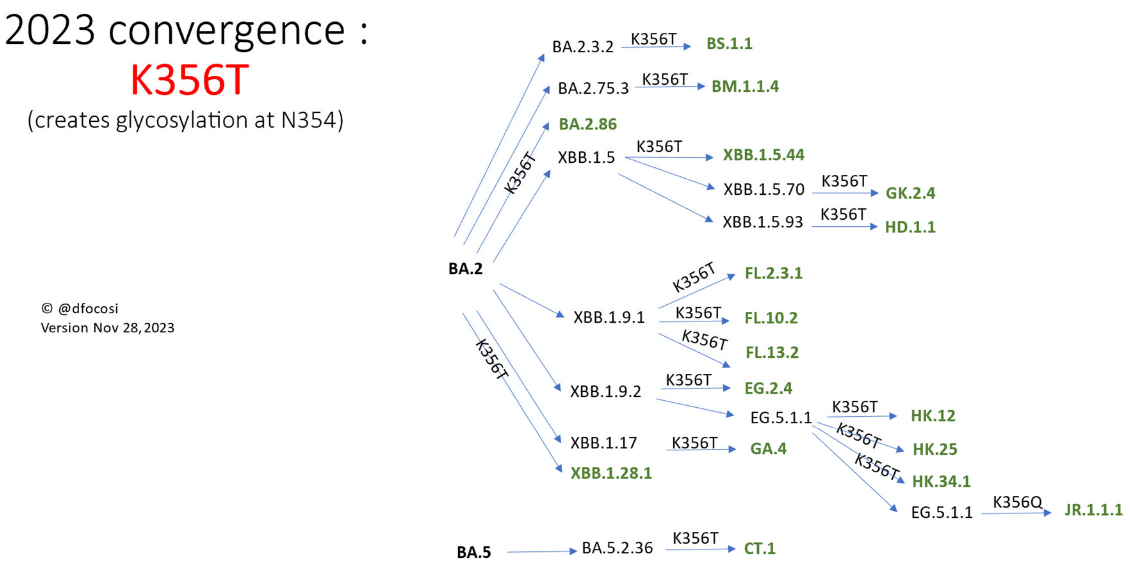

Figure S1. Summary of independent S:K356T acquisition events in designated PANGOLIN lineages.

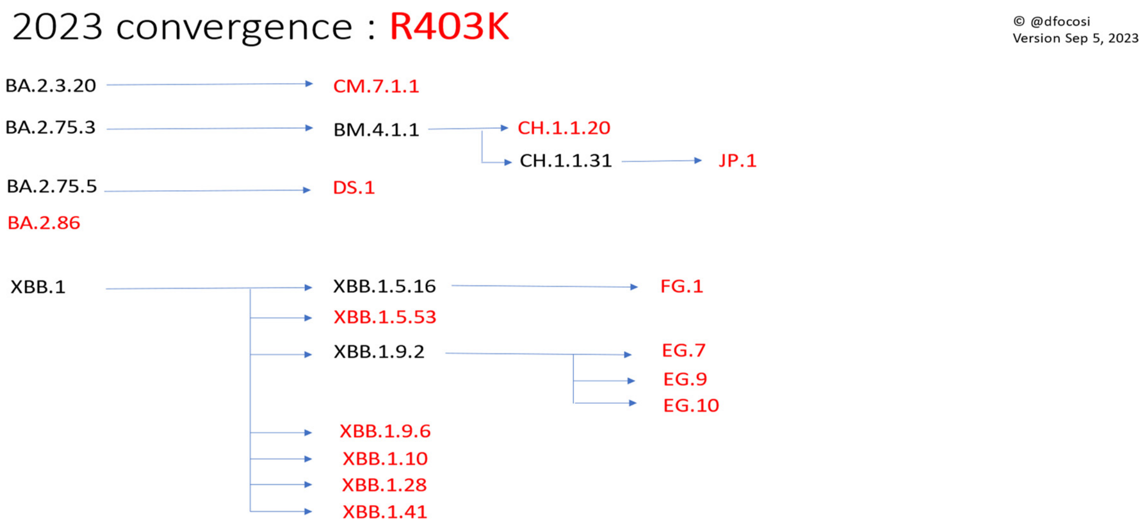

Figure S2. Summary of S:R403K acquisition events in designated PANGOLIN lineages.

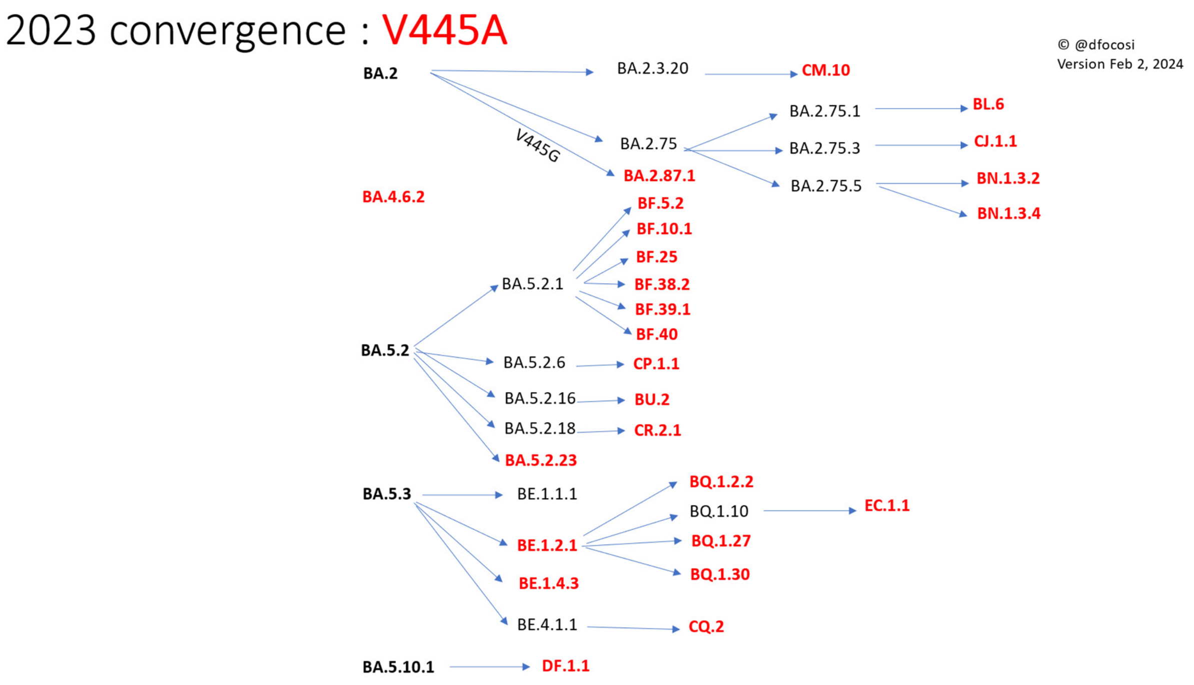

Figure S3. Summary of independent S:V445A acquisition events in designated PANGOLIN lineages.

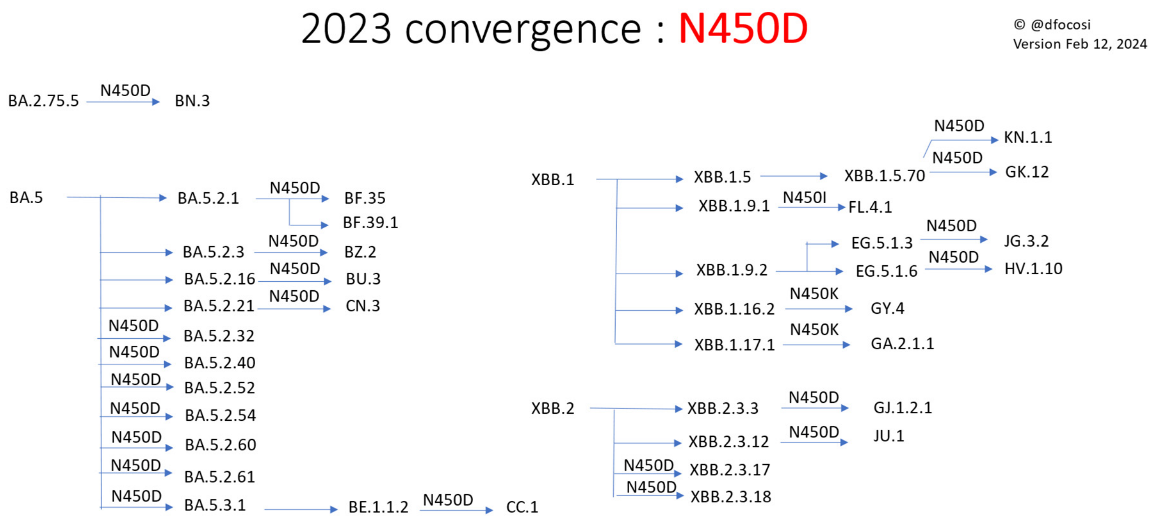

Figure S4. Summary of independent S:N450D acquisition events in designated PANGOLIN lineages.

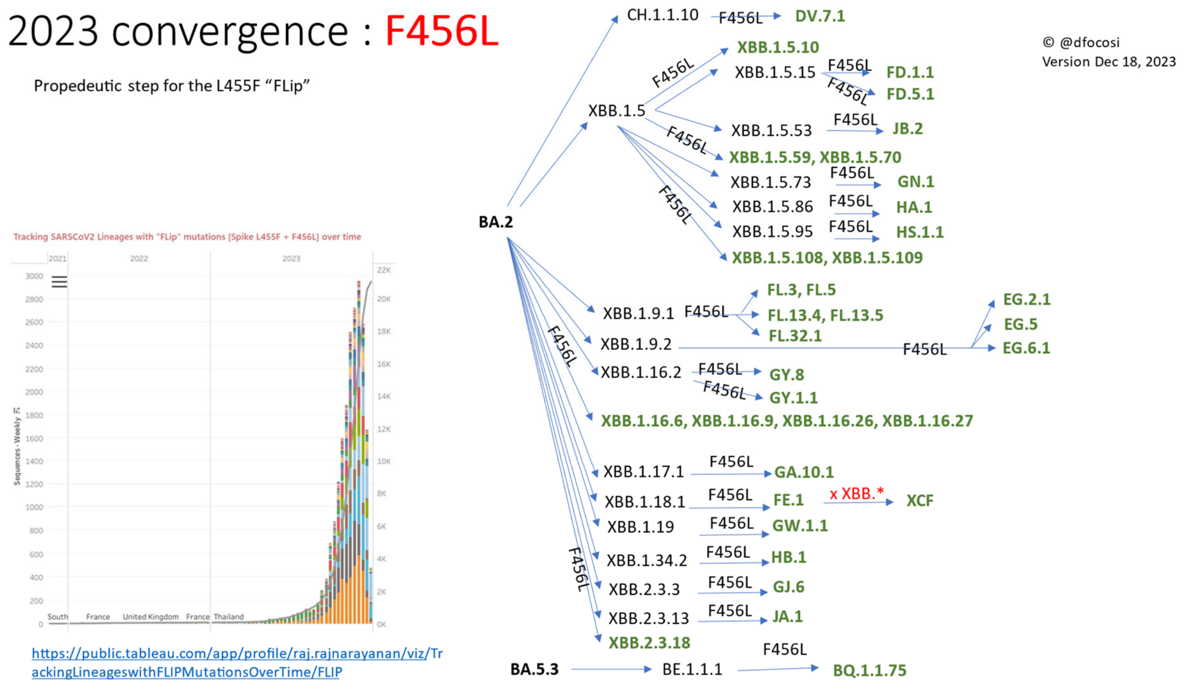

Figure S5. Summary of independent S:F456L acquisition events in designated PANGOLIN lineages.

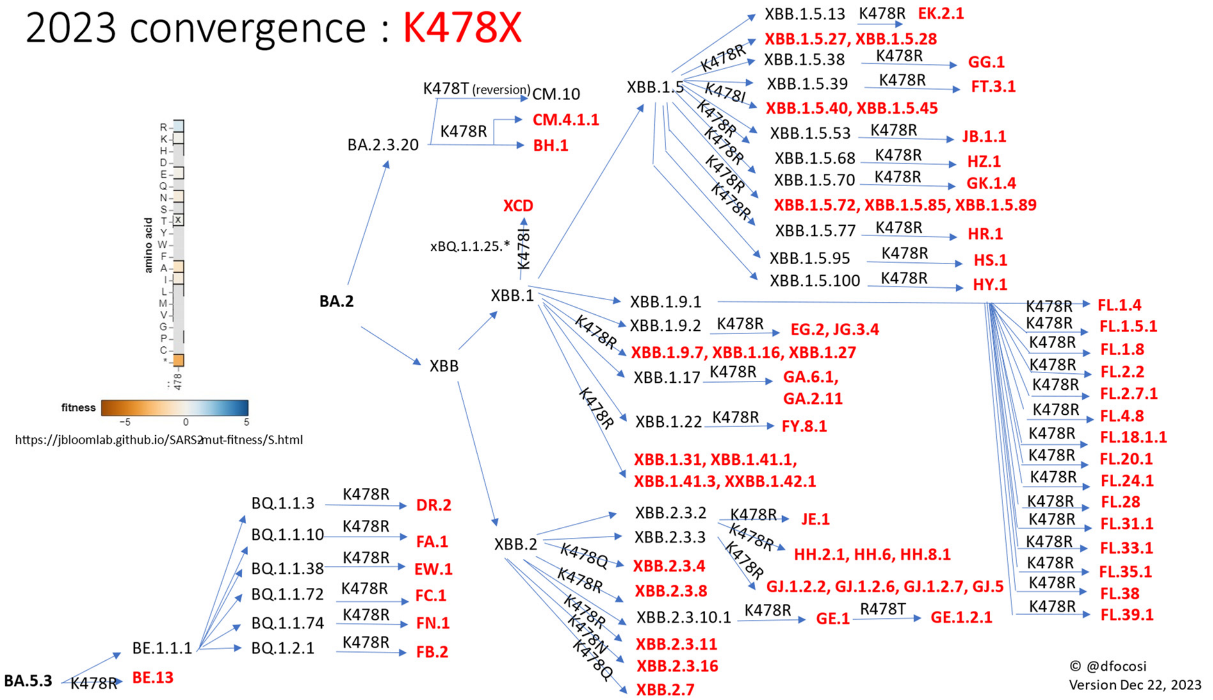

Figure S6. Summary of independent S:K478X acquisition events in designated PANGOLIN lineages.

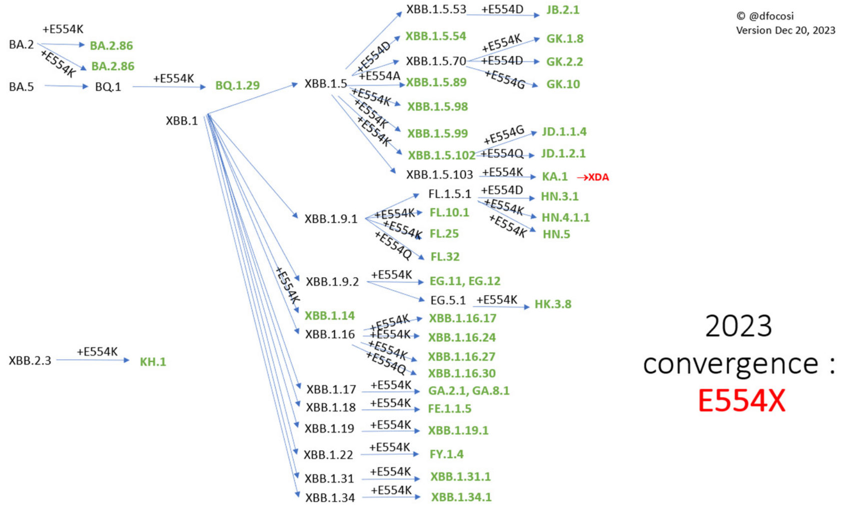

Figure S7. Summary of independent S:E554K acquisition events in designated PANGOLIN lineages.

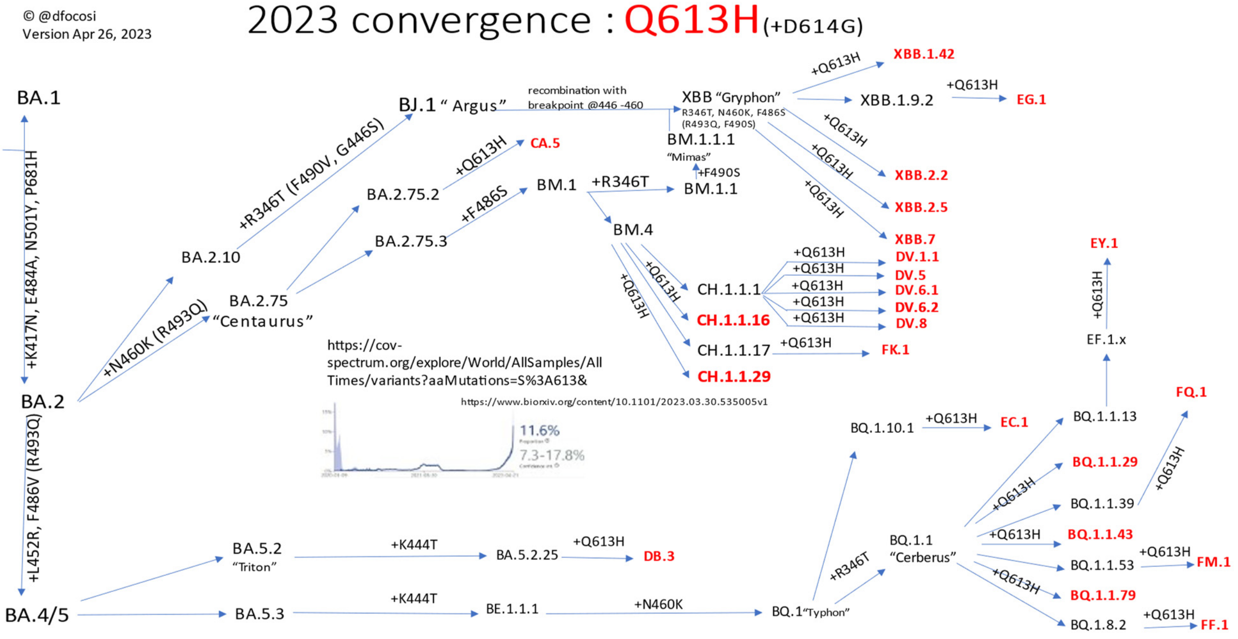

Figure S8. Summary of independent S:Q613H acquisition events in designated PANGOLIN lineages.

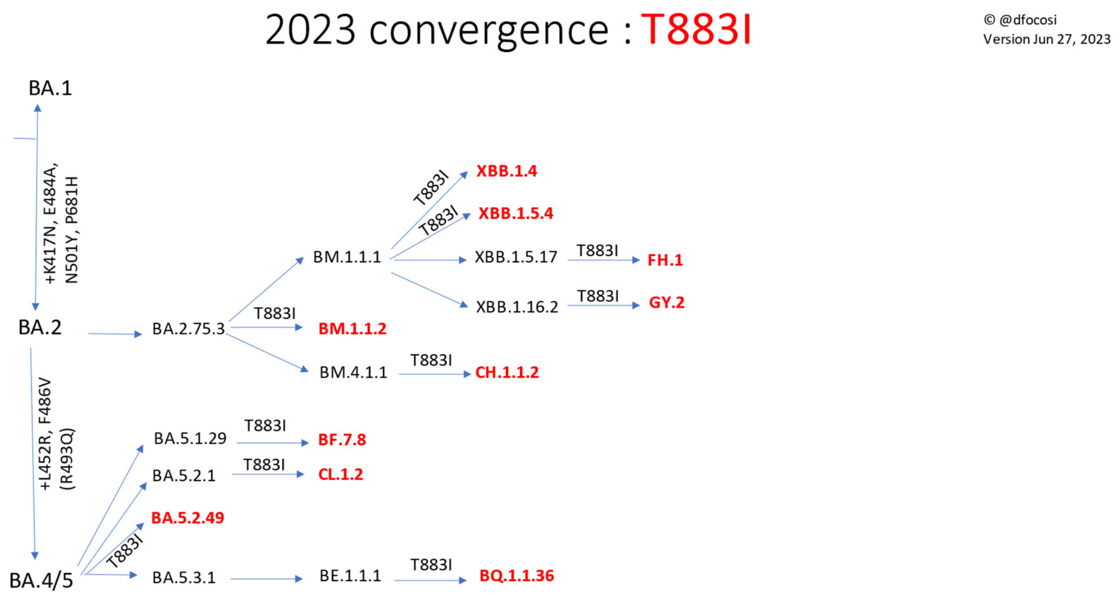

Figure S9. Summary of independent S:T883I acquisition events in designated PANGOLIN lineages.
